# Effects of aerobic exercise combined with blood flow restriction on physical fitness and mental health of high school students

**DOI:** 10.3389/fpsyg.2025.1654855

**Published:** 2025-09-15

**Authors:** Xianglong Jiang, Tongtong Che

**Affiliations:** School of Physical Education, Qingdao University, Qingdao, China

**Keywords:** aerobic exercise, blood flow restriction training, high school students, physical fitness, mental health

## Abstract

**Objective:**

This study aims to investigate the combined effects of aerobic exercise and Blood Flow Restriction (BFR) on physical fitness and mental health among high school students, providing empirical evidence for the reform and optimization of school physical education programs.

**Methods:**

A pre-post comparative design was adopted involving 58 high school students aged between 16 and 18 years, randomly assigned to the Teaching Intervention Group (aerobic exercise combined with BFR *n* = 19), Comparison Group (aerobic exercise only, *n* = 19), and Control Group (no additional intervention, *n* = 20). The intervention lasted 12 weeks, with two 45-min training sessions per week. Physical fitness indicators, including vital capacity, endurance run, 50-meter sprint, standing long jump, and sit-and-reach test, were evaluated according to the National Student Physical Fitness Standards. Mental health was assessed using the Chinese Middle School Students’ Mental Health Inventory (MMHI-60).

**Results:**

Both the Teaching Intervention Group and Comparison Group demonstrated significant improvements in all physical fitness indicators (ps < 0.001). However, compared to the Control Group, the Teaching Intervention Group showed significantly greater improvements in endurance run (*p* < 0.05, d = 0.71), 50-meter sprint (*p* < 0.05, d = 0.86), and standing long jump (*p* < 0.001, *d* = 0.96), along with higher improvements in sit-and-reach test (*d* = 2.87). Regarding mental health, the Teaching Intervention Group exhibited significantly superior effects in core indicators such as anxiety (*d* = 1.51), depression (*d* = 1.72), and academic stress (*d* = 1.43) compared to the Comparison Group, with a significant difference observed specifically in anxiety (*p* < 0.05). Correlation analysis indicated ten significant associations between physical fitness and mental health indicators in the Teaching Intervention Group, whereas only two significant correlations were identified in the Comparison Group.

**Conclusion:**

Aerobic exercise combined with Blood Flow Restriction effectively improves physical fitness and mental health in high school students, demonstrating greater efficacy compared to aerobic exercise alone. This combined approach also reveals a stronger synergistic promotion of physical and mental health. These findings provide new intervention perspectives for secondary school physical education, supporting BFRT as an effective instructional method in school physical education curricula.

## 1 Introduction

Adolescence is a critical period for individual physiological, psychological, and social behavioral development. In particular, high school students are undergoing pivotal phases characterized by significant physical growth, cognitive advancement, and the formation of social adaptability (Lin and Li, 2005). However, with increasing academic stress, lifestyle changes, and the growing complexity of the social environment, adolescents are facing increasingly severe physical and psychological health issues. High incidences of psychological problems, particularly anxiety and depression, present unprecedented health challenges (Zhu et al., 2021). The adolescent stage is not only crucial for physical development but also marked by heightened psychological vulnerability. Thus, effectively promoting adolescents’ physical and mental health within the educational system has become a central concern in contemporary school education.

School physical education courses, as an integral component of the national health promotion strategy, have long been committed to enhancing students’ physical fitness and psychological adaptability through scientifically structured curricula and diverse exercise modalities. Among these modalities, aerobic exercises (such as running and jump rope) are widely implemented in physical education classes due to their convenience and adaptability. Research has confirmed that aerobic exercise effectively improves adolescents’ cardiopulmonary function and endurance, while also positively reducing anxiety, alleviating stress, and enhancing mood (van Sluijs et al., 2021). However, traditional aerobic instruction has limitations in enhancing muscle strength, rapid reaction capacity, and psychological regulation (Wang et al., 2023a), prompting educational reformers to continually seek more challenging and effective instructional methods.

Recently, Blood Flow Restriction Training (BFRT), also known as KAATSU Training, combined with aerobic exercise has gradually attracted attention in health promotion research. BFRT partially restricts blood flow to the limbs, thereby intensifying metabolic stress and physiological load during exercise. Even at lower intensities, it achieves effects similar to high-intensity training, significantly improving muscle strength and endurance (Dong et al., 2025). Integrating BFR into aerobic exercise instruction not only enhances cardiopulmonary function but also potentially strengthens the teaching effectiveness for strength and endurance training, comprehensively promoting adolescent physical fitness. As training loads increase, BFRT can also stimulate adolescents’ physiological adaptation mechanisms, improve exercise performance, alleviate psychological stress, and enhance psychological resilience (Silva et al., 2021). Although BFRT has been extensively used in professional athletic and rehabilitation settings, its instructional application in secondary school physical education classes, particularly regarding its comprehensive effects on physical fitness and psychological health when combined with aerobic exercises, is still in the preliminary stage.

Therefore, this study employs high school physical education classes as an implementation platform, establishing three distinct instructional conditions: an Teaching Intervention Group incorporating aerobic exercise with Blood Flow Restriction, a Comparison Group involving aerobic exercise alone, and a Control Group with no additional intervention. By comparing the outcomes of these instructional approaches on physical fitness (such as muscle strength and endurance) and psychological health (including anxiety, depression, and academic stress), the study aims to provide empirical evidence and instructional references for optimizing physical education curricula and enhancing their role in health education. In line with these objectives, the following hypotheses are proposed: (1) aerobic exercise combined with blood flow restriction will yield greater improvements in both physical fitness and mental health compared with aerobic exercise alone and no intervention; and (2) aerobic exercise alone will provide moderate benefits relative to the control group, but to a lesser extent than the combined intervention.

## 2 Participants and methods

### 2.1 Participants

This study selected students aged 16 to 18 from a high school in Qingdao, aiming to explore the effects of different instructional activities on students’ physical fitness and psychological health. To ensure scientific rigor and feasibility, a total of 60 students were recruited and stratified randomly by gender and age into three instructional groups: the group receiving aerobic exercise combined with Blood Flow Restriction Training (Teaching Intervention Group), the group receiving aerobic exercise alone (Comparison Group), and the group following routine physical activity without additional intervention (Control Group), with 20 students in each group initially.

All participants volunteered for the study and signed informed consent forms after being fully informed of the study’s objectives, procedures, and potential risks. Group allocation was performed using a computer-generated random number table to ensure scientific validity and unbiased assignment. During the intervention period, two participants (one from the Teaching Intervention Group and one from the Comparison Group) withdrew for personal reasons. As a result, the final sample sizes were 19 participants in the Teaching Intervention Group, 19 participants in the Comparison Group, and 20 participants in the Control Group. In total, 58 participants successfully completed the full intervention. Training compliance was high, with an average attendance rate of 95.2% in both the Teaching Intervention Group and the Comparison Group. Data from individuals who missed more than 10% of the total sessions were excluded from the final analysis.

This study was approved by the Ethics Committee of Qingdao University Medical College (Approval No.: QDU-HEC-2025355). All participants and their legal guardians were clearly informed about the study’s purpose, content, and potential risks and voluntarily participated based on informed consent. Throughout the research process, privacy protection and ethical standards were strictly upheld. All personal information and test data were stored using coded identifiers to ensure confidentiality and data security.

Inclusion criteria: (1) high school students aged 16–18; (2) physically healthy with no serious chronic diseases (e.g., cardiovascular or neurological disorders); (3) no history of long-term medication use and no previous participation in other physical or psychological health interventions; (4) able to attend all training sessions punctually.

Exclusion criteria: (1) presence of serious chronic diseases or movement disorders; (2) participation in systematic physical training or interventions within the past month; (3) inability to ensure full participation (e.g., withdrawal or frequent absences).

### 2.2 Experimental design and training protocol

#### 2.2.1 Experimental design

This study employed a pretest-posttest comparison design combined with randomized group assignment to examine the effects of different instructional activities. The experimental process consisted of three phases: pretest, instructional intervention, and posttest. The pretest was conducted 1 week before the intervention (Week 0), and the posttest was administered 1 week after the intervention concluded (Week 13) to evaluate the effects of the instructional activities. A mid-intervention assessment was conducted at the end of Week 6 to inform potential training plan adjustments; however, the results of this assessment were not included in the final analysis.

The intervention lasted for 12 weeks. The Teaching Intervention Group and the Comparison Group participated in training sessions twice per week, with each session lasting 45 min. The Control Group maintained their usual lifestyle without any additional intervention. Their daily activity levels were monitored weekly using activity logs to ensure that their physical activity remained stable and did not undergo significant changes.

To control for confounding variables, all participants were required to adhere to the following guidelines throughout the study: (1) avoid strenuous physical activity within 24 h prior to testing; (2) refrain from consuming caffeinated foods or beverages on the day of testing; (3) maintain regular dietary and sleep habits; and (4) refrain from participating in any other organized physical training outside of the experimental program. Testing conditions were standardized, with an ambient temperature of 22 ± 2°C and relative humidity of 50 ± 5%. All group assessments were scheduled within the same time frame (2:00–4:00 p.m.).

#### 2.2.2 Training protocol

Training interventions were conducted twice per week, each session lasting 45 min and consisting of a warm-up (10 min), main training (30 min), and cool-down with stretching (5 min). The intervention was divided into two stages: a low-intensity aerobic phase (Weeks 1–6) and a moderate-intensity aerobic phase (Weeks 7–12). According to the guidelines by Vanhees et al. (2012), the target heart rate was maintained at 45–54% of the individual’s maximum heart rate (calculated by the formula: 220 − age) during the low-intensity phase and 55–69% during the moderate-intensity phase.

**Figure 1:**
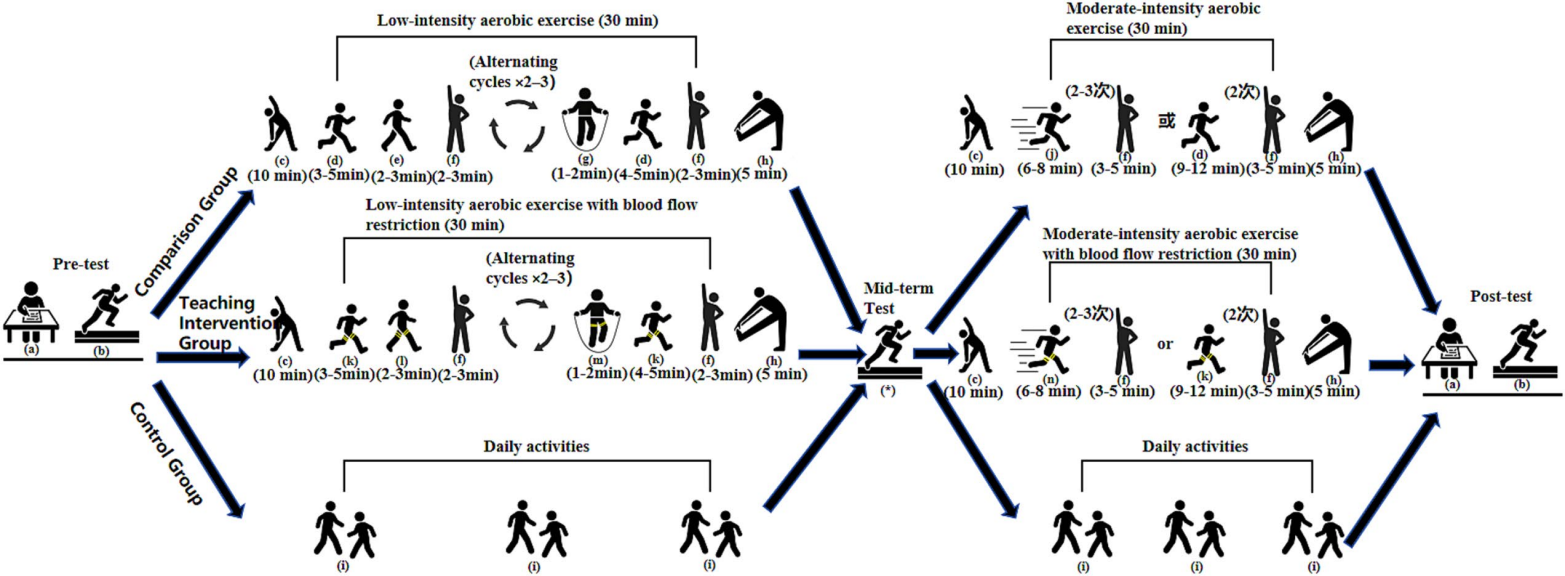
Flowchart of the experimental design and training program. a: Completion of psychological health questionnaire; b: Physical fitness assessment; c: Warm-up activities; d: Jogging; e: Brisk walking; f: Rest (or low-intensity recovery activity); g: Rope skipping; h: Cool-down and stretching; i: Daily routine activities; j: Moderate-paced running; k: Jogging with lower-limb BFR; l: Brisk walking with lower-limb BFR; m: Rope skipping with lower-limb BFR; n: Moderate-paced running with lower-limb BFR;*: Mid-term physical fitness assessment (end of Week 6; used solely for training adjustment, not included in final data analysis).

Both the Teaching Intervention Group and the Comparison Group participated in the aerobic exercise program. The training content was based on the adolescent-oriented design proposed by Ortiz Sánchez and Hidalgo Morales (2024), incorporating aerobic activities such as jogging, brisk walking, and rope skipping. During the low-intensity phase (Weeks 1–6), an intermittent training model was used, e.g., jogging for 3–5 min followed by brisk walking for 2–3 min, or rope skipping for 1–2 min followed by jogging for 4–5 min, with each session including 2–3 such sets. In the moderate-intensity phase (Weeks 7–12), the proportion of continuous training increased, with longer bouts such as medium-paced running for 6–8 min or continuous jogging for 9–12 min, supplemented by intermittent training to improve cardiopulmonary function and endurance (see Figure 1).

On top of the identical aerobic training program conducted by the Comparison Group, the Teaching Intervention Group also engaged in lower limb Blood Flow Restriction using a portable smart pressure control device (Yidongkang Intelligent Pressure Training Instrument, Model BZXNJY-01, China). The device has a pressure range of 0–300 mmHg and is capable of automatically displaying and regulating cuff pressure in real time during exercise. Pressure parameters were set according to device guidelines and based on studies by Wei et al. (2019) and Abe et al. (2010). These studies typically set the adult BFR pressure to 160 mmHg at the start, gradually increasing to 210 mmHg for effective training. These parameters were adjusted to account for the physical characteristics and developmental stage of adolescents, reduced by approximately 20–30% from adult standards to ensure safety. Specifically, during the low-intensity phase, band pressure was set to 40–60 mmHg with cuff inflation pressure at 120 mmHg; during the moderate-intensity phase, band pressure was set to 60–80 mmHg with inflation pressure at 140 mmHg. Pressure cuffs were placed at the proximal thigh near the groin area, with a width of 5 cm. Pressure was progressively increased from the low- to moderate-intensity phase to follow the principle of progressive overload and to allow participants to adapt to the physiological stress of BFR.

Following recommendations by Hughes et al. (2017) and Patterson et al. (2019), an intermittent pressure mode was adopted rather than continuous occlusion. In the low-intensity phase (Weeks 1–6), each bout of occlusion lasted approximately 5–8 min followed by 2–3 min of rest; in the moderate-intensity phase (Weeks 7–12), occlusion durations extended to 6–12 min with 3–5 min of rest. Given the adolescent population, safety during BFR was a primary concern. Accordingly, several precautionary measures were implemented: (1) subjective exertion was monitored in real-time using the Borg Rating of Perceived Exertion Scale (6–20), maintaining scores within the range of 12–16; (2) heart rate was continuously monitored via heart rate belts to ensure it did not exceed 70% of the individual’s maximum heart rate; and (3) in case of discomfort (e.g., dizziness, nausea, or severe pain), pressure was immediately released and training was suspended. No serious adverse events were observed during the intervention. A few participants (*n* = 3) reported mild discomfort (e.g., limb numbness) during the initial phase of Blood Flow Restriction Training (BFR), which was promptly alleviated through pressure adjustments; their data were included in the analysis, as the discomfort was transient, did not result in long-term effects, and did not necessitate withdrawal from the study. In addition, after each training session, participants were systematically screened for potential adverse effects (e.g., numbness, dizziness, disproportionate pain) using a standardized checklist. Through these safety measures and monitoring procedures, no severe adverse events (such as rhabdomyolysis or venous thrombosis) were observed throughout the intervention, with all training sessions conducted under the joint supervision of experienced physical education teachers and certified exercise rehabilitation professionals to ensure both safety and efficacy.

### 2.3 Experimental indicators

#### 2.3.1 Physical fitness indicators

This study adopted relevant assessment items from the National Physical Fitness Standards for Students (Revised 2014) to evaluate participants’ physical fitness levels. The evaluation covered multiple dimensions, including body morphology (Body Mass Index), physiological function (Vital Capacity), and physical qualities such as speed, endurance, strength, and flexibility. This testing system has been widely applied in physical education among Chinese adolescents and has demonstrated strong reliability and validity (Zhang, 2014).

To enhance the sensitivity of detecting changes in measured outcomes, the original scoring criteria (which used 5-point intervals) were refined using an equidistant scale. Specifically, the original 5-point score ranges were subdivided into 1-point intervals. A linear interpolation and rounding method was applied to define the corresponding performance range for each single-point score. The refined scoring scale was reviewed and confirmed by three physical education experts. Importantly, this adjustment did not alter the original testing procedures or content validity but significantly improved the precision and discriminative power of the evaluation, thereby enhancing its ability to capture subtle changes in participants’ physical indicators.

The specific test items included the 50-meter Sprint, the 1,000-meter Endurance Run (for boys) or the 800-meter Endurance Run (for girls), the Standing Long Jump, Vital Capacity, and the Sit-and-Reach Test. These items comprehensively reflect multiple aspects of students’ physical fitness. All assessments followed the procedures and scoring standards set by the National Physical Fitness Standards for Students (Revised 2014) to ensure scientific rigor and fairness.

All tests were conducted in an indoor gymnasium under controlled environmental conditions (temperature 22 ± 2°C; relative humidity 50 ± 5%) and were uniformly scheduled between 2:00 and 4:00 p.m. to minimize the influence of environmental factors and circadian rhythms on performance. Three physical education teachers who had received specialized training and passed inter-rater reliability tests (ICC > 0.90) administered the tests to ensure accuracy and consistency. A standardized 10-min warm-up was conducted before each session to ensure proper muscle preparation and prevent injury.

#### 2.3.2 Psychological health indicators

This study utilized the Mental Health Inventory of Middle School Students (MMHI-60) to assess participants’ psychological well-being. Developed by Wang et al. (1997), the MMHI-60 is specifically designed to evaluate the mental health of Chinese middle school students and has demonstrated strong reliability and validity. It has been widely applied in psychological health research among Chinese adolescents and has shown good internal consistency and stability across multiple studies. The Cronbach’s *α* coefficient for the total scale ranges from 0.651 to 0.857, while those for individual subscales range from 0.6341 to 0.8726. The test–retest reliability ranges from 0.716 to 0.905, indicating good applicability and stability for use with adolescent populations. Recent studies (e.g., Tan et al., 2023; Zhai et al., 2024) have further confirmed its validity and supported its broad applicability across various populations.

The MMHI-60 consists of 60 items covering 10 dimensions: Obsessive-Compulsive Symptoms, Paranoia, Hostility, Interpersonal Sensitivity, Depression, Anxiety, Academic Stress, Maladjustment, Emotional Instability, and Psychological Imbalance. These dimensions comprehensively reflect the spectrum of potential psychological issues among middle and high school students. The inventory adopts a 5-point Likert scale (1 = strongly disagree, 5 = strongly agree), with some items reverse scored. Each dimension score is calculated as the average of all items within that domain, and the total score is the sum of all item scores. Higher scores indicate greater psychological distress, whereas lower scores reflect better psychological health.

The MMHI-60 was administered in a group-based format under the guidance of trained graduate students in psychology. Assessments were conducted in quiet, distraction-free classrooms, and completion time ranged from 20 to 25 min to ensure participants could fully concentrate on the task. Upon completion, two trained research staff members independently entered and cross-checked the data to ensure accuracy. To improve the authenticity of responses, psychological assessments were conducted on days different from the physical fitness tests. Participants were clearly informed that the evaluation results were solely for research purposes and would not affect their academic performance, thus minimizing the influence of social desirability bias.

### 2.4 Statistical analysis

Data were organized and statistically analyzed using Excel 2016 and SPSS version 27.0. All results are presented as mean ± standard deviation (M ± SD). The analysis consisted of both descriptive and inferential statistics. For inferential analysis, a Two-way Repeated Measures ANOVA was conducted, with instructional condition (Teaching Intervention Group, Comparison Group, Control Group) as the between-subjects factor and time point (pretest, posttest) as the within-subjects factor, to evaluate the effects of different instructional interventions on students’ physical fitness and psychological health.

Prior to analysis, the data were subjected to a normality check using the Shapiro–Wilk test to determine the suitability of parametric testing. For data that did not meet the assumption of normality, logarithmic transformation was applied. The Mauchly’s test of sphericity was also performed to test the assumption of sphericity. When this assumption was violated, degrees of freedom were adjusted using the Greenhouse-Geisser correction.

The primary focus of the ANOVA was to examine the main effects of group and time, as well as their interaction. If a significant interaction effect was observed, simple effects analyses were conducted to compare pretest and posttest results within each group, as well as between-group differences at each time point. Bonferroni correction was applied for all multiple comparisons to control the risk of Type I error.

To evaluate the practical significance of the instructional intervention, partial eta squared (*η*^2^) values were reported as measures of effect size, and interpreted using Cohen’s guidelines: *η*^2^ = 0.01 (small), *η*^2^ = 0.06 (medium), and *η*^2^ = 0.14 (large). Additionally, Cohen’s d was calculated for between-group comparisons to quantify effect sizes, with *d* = 0.2 indicating a small effect, *d* = 0.5 a medium effect, and *d* = 0.8 a large effect.

Additionally, the relationship between changes in physical fitness and psychological health indicators (calculated as the difference between posttest and pretest values) was examined using Pearson’s correlation coefficient for normally distributed data or Spearman’s rank correlation coefficient for non-normally distributed data. The significance level for all statistical tests was set at *p* < 0.05.

## 3 Results

### 3.1 Changes in physical fitness indicators

Results from the repeated measures ANOVA revealed highly significant main effects of time across all physical fitness indicators (ps < 0.001), indicating that overall improvements occurred following the instructional interventions. The main effects of group were not significant for Vital Capacity, Endurance Run, 50-meter Sprint, Standing Long Jump, and Sit-and-Reach Test (ps > 0.05), suggesting no overall group differences. However, all indicators showed highly significant group × time interaction effects (ps < 0.001), indicating that the intervention effects varied among the different instructional groups.

Further within-group comparisons showed that Vital Capacity significantly increased in both the Teaching Intervention Group (*p* < 0.001, *d* = 1.65, large effect size) and the Comparison Group (*p* < 0.001, *d* = 1.42, large effect size), while no significant change was observed in the Control Group. For the Endurance Run, both the Teaching Intervention Group (*p* < 0.001, *d* = 1.96, very large effect size) and the Comparison Group (*p* < 0.001, *d* = 5.09, very large effect size) demonstrated significant improvements, with no significant change in the Control Group. Regarding the 50-meter Sprint, significant improvements were also observed in the Teaching Intervention Group (*p* < 0.001, *d* = 1.22, very large effect size) and the Comparison Group (*p* < 0.001, *d* = 2.56, very large effect size), but not in the Control Group. For the Standing Long Jump, both the Teaching Intervention Group (*p* < 0.001, *d* = 2.64, very large effect size) and the Comparison Group (*p* < 0.001, *d* = 2.89, very large effect size) showed significant improvements, whereas the Control Group did not. For the Sit-and-Reach Test, a significant improvement was observed in the Teaching Intervention Group (*p* < 0.001, *d* = 2.87, very large effect size); the Comparison Group also showed a statistically significant but moderate improvement (*p* < 0.05, *d* = 0.53, medium effect size), while no change was found in the Control Group.

**Figure 2:**
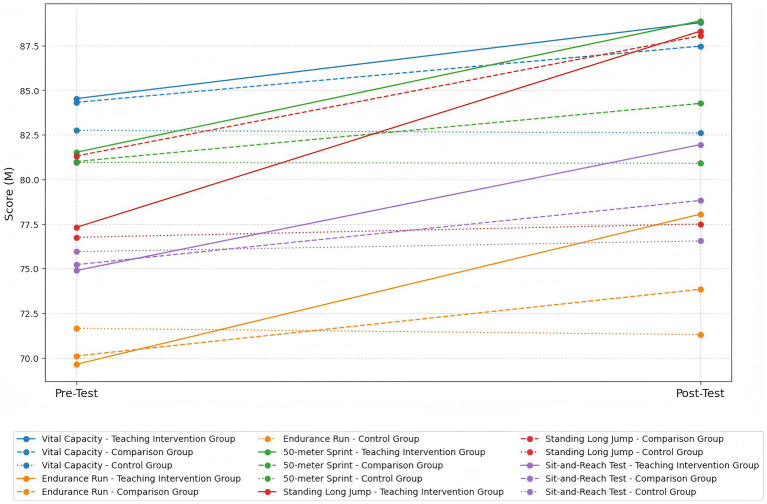
Trends in the average scores of physical fitness indicators before and after the intervention across the three groups.

Results from between-group multiple comparisons with Bonferroni correction indicated that the Teaching Intervention Group outperformed the Control Group in the Endurance Run (*p* < 0.05, *d* = 0.71, medium effect size), 50-meter Sprint (*p* < 0.05, *d* = 0.86, large effect size), and Standing Long Jump (*p* < 0.001, *d* = 0.96, large effect size). The Comparison Group showed a significant difference from the Control Group only in the Standing Long Jump (*p* < 0.05, *d* = 0.88, large effect size). No other between-group differences reached statistical significance (ps > 0.05), as presented in Table 1 and Figure 2.

**Table 1:** Changes in physical fitness indicators before and after the intervention and results of repeated measures ANOVA.

Indicator	Group	Pretest (M ± SD)	Posttest (M ± SD)	Main Effect (partial η^2^)		F [group] × [time]
				Group	Time	
Vital Capacity	Teaching Intervention Group	84.53 ± 11.85	88.79 ± 10.93	0.634 (0.023)	80.460*** (0.594)	24.444*** (0.471)
	Comparison Group	84.32 ± 12.07	87.47 ± 11.83			
	Control Group	82.75 ± 12.13	82.60 ± 11.95			
Endurance Run	Teaching Intervention Group	69.63 ± 5.77	78.05 ± 5.59	0.409 (0.015)	87.437*** (0.614)	36.515*** (0.57)
	Comparison Group	70.10 ± 4.51	73.84 ± 3.98			
	Control Group	71.65 ± 13.89	71.3 ± 12.31			
50-meter Sprint	Teaching Intervention Group	81.52 ± 11.01	88.89 ± 9.43	0.378 (0.507)	56.577*** (0.507)	21.079*** (0.434)
	Comparison Group	81 ± 9.71	84.26 ± 10.12			
	Control Group	80.95 ± 8.75	80.9 ± 9.17			
Standing Long Jump	Teaching Intervention Group	77.31 ± 9.67	88.31 ± 8.17	2.415 (0.081)	228.669*** (0.806)	53.827*** (0.662)
	Comparison Group	81.31 ± 10.17	88.05 ± 9.97			
	Control Group	76.75 ± 14.51	77.5 ± 13.74			
Sit-and-Reach	Teaching Intervention Group	74.89 ± 7.07	81.95 ± 8.78	0.332 (0.012)	48.649*** (0.469)	11.922*** (0.302)
	Comparison Group	75.22 ± 10.33	78.82 ± 10.33			
	Control Group	75.95 ± 9.25	76.55 ± 9.37			

*p < 0.05, **p < 0.01, ***p < 0.001.

### 3.2 Changes in psychological health indicators

Results from the repeated measures ANOVA showed a significant main effect of time for Maladjustment (*p* < 0.05), and very significant time effects for Hostility, Obsessive-Compulsive Symptoms, and Interpersonal Sensitivity (ps < 0.01). Extremely significant time effects were observed for Anxiety, Emotional Instability, Academic Stress, and Depression (ps < 0.001), whereas the time effects for Paranoia and Psychological Imbalance were not significant (ps > 0.05). A very significant main effect of group was found only for the Anxiety factor (*p* < 0.01), while no significant group effects were observed for other indicators (ps > 0.05). Significant group × time interaction effects were observed for Hostility and Maladjustment (ps < 0.05), a very significant interaction for Emotional Instability (*p* < 0.01), and extremely significant interactions for Anxiety, Academic Stress, and Depression (ps < 0.001), indicating that the intervention effects varied among the groups.

Further within-group comparisons revealed significant improvements in the Teaching Intervention Group on Hostility (*p* < 0.01, *d* = 0.69, medium effect size), Emotional Instability (*p* < 0.01, *d* = 0.70, medium effect size), Maladjustment (*p* < 0.01, *d* = 0.83, large effect size), Academic Stress (*p* < 0.001, *d* = 1.43, very large effect size), and Depression (*p* < 0.001, *d* = 1.72, very large effect size), with the most prominent improvement observed in Anxiety (*p* < 0.001, *d* = 1.51, very large effect size). The Comparison Group also demonstrated significant improvements in Hostility (*p* < 0.01, *d* = 0.73, medium effect size), Maladjustment (*p* < 0.01, *d* = 0.81, large effect size), and Depression (*p* < 0.01, *d* = 0.75, large effect size). Moreover, it showed extremely significant improvements in Anxiety (*p* < 0.001, *d* = 1.21, very large effect size), Emotional Instability (*p* < 0.001, *d* = 1.83, very large effect size), and Academic Stress (*p* < 0.001, *d* = 1.51, very large effect size). The Control Group did not exhibit any significant changes across all psychological health indicators.

**Figure 3:**
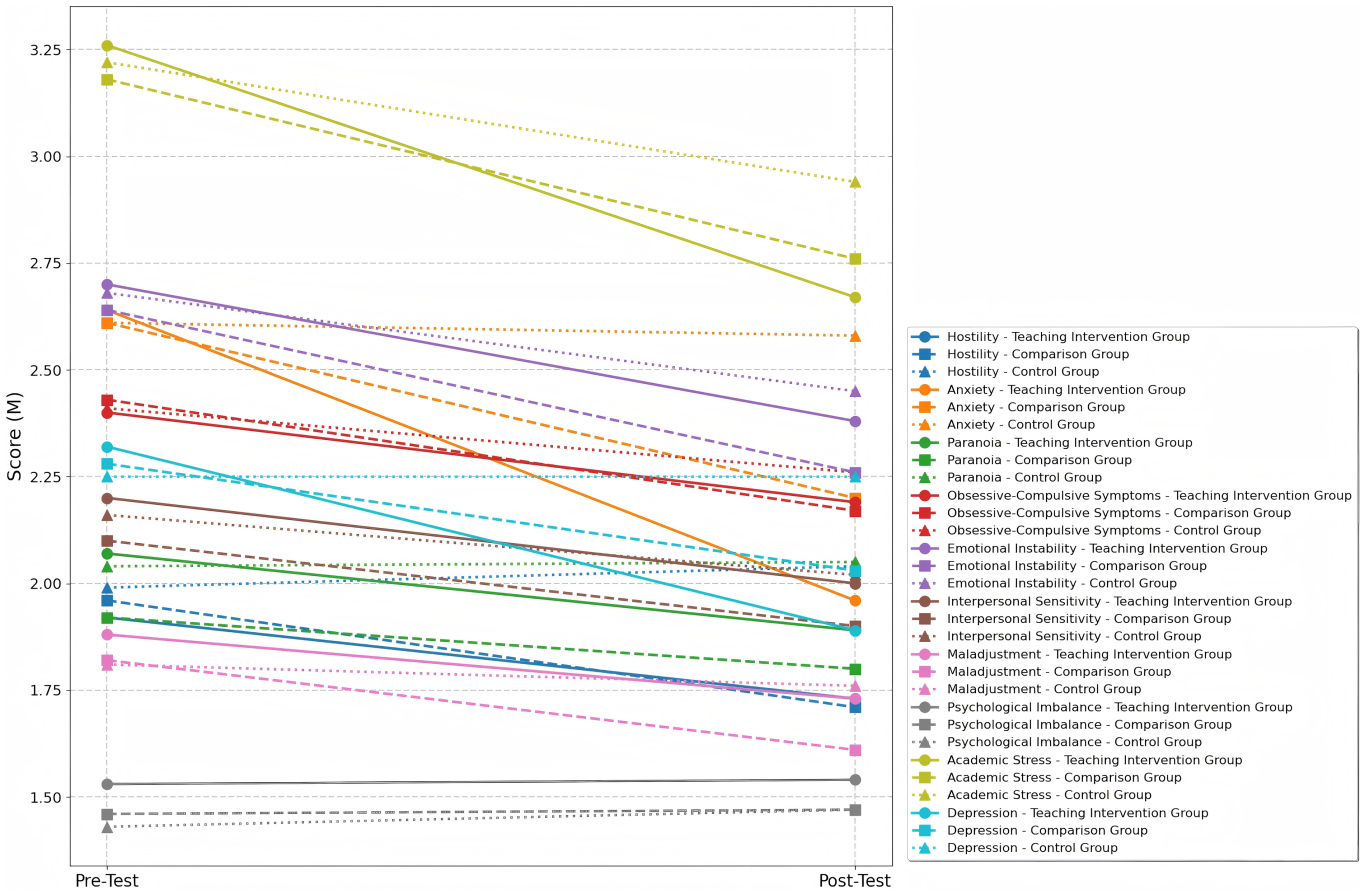
Trends in the average scores of mental health indicators before and after the intervention across the three groups.

Results of between-group multiple comparisons (Bonferroni correction) indicated that the Teaching Intervention Group significantly outperformed the Control Group in Hostility (*p* < 0.05, *d* = −0.91, large effect size), Emotional Instability (*p* < 0.01, *d* = −1.18, large effect size), and Depression (*p* < 0.001, *d* = −1.46, very large effect size). Extremely significant differences were also observed in Anxiety (*p* < 0.001, *d* = 2.09, very large effect size) and Academic Stress (*p* < 0.001, *d* = 1.62, very large effect size). The Comparison Group showed significant differences from the Control Group in Hostility (*p* < 0.05, *d* = −0.92, large effect size) and extremely significant differences in Anxiety (*p* < 0.001, *d* = 1.30, very large effect size), Emotional Instability (*p* < 0.001, *d* = −1.43, very large effect size), and Academic Stress (*p* < 0.001, *d* = 1.21, very large effect size). Between the Teaching Intervention Group and the Comparison Group, only Anxiety showed a significant difference (*p* < 0.05, *d* = 1.00, large effect size), while no significant differences were found between these two groups on the other indicators (ps > 0.05), as presented in Table 2 and Figure 3.

**Table 2:** Changes in mental health indicators in each group before and after the intervention and results of repeated measures ANOVA.

Indicator	Group	Pretest (M ± SD)	Posttest (M ± SD)	Main Effect (partial η^2^)		F [group] × [time]
				Group	Time	
Hostility	Teaching Intervention Group	1.92 ± 0.32	1.73 ± 0.28	0.128 (0.72)	7.845** (0.125)	3.939* (0.125)
	Comparison Group	1.96 ± 0.39	1.71 ± 0.32			
	Control Group	1.99 ± 0.86	2.04 ± 0.39			
Anxiety	Teaching Intervention Group	2.64 ± 0.39	1.96 ± 0.23	5.817** (0.175)	60.208*** (0.523)	15.996*** (0.368)
	Comparison Group	2.61 ± 0.31	2.2 ± 0.23			
	Control Group	2.61 ± 0.41	2.58 ± 0.34			
Paranoia	Teaching Intervention Group	2.07 ± 0.37	1.89 ± 0.34	1.838 (0.063)	3.918(0.066)	1.271 (0.044)
	Comparison Group	1.92 ± 0.27	1.80 ± 0.31			
	Control Group	2.04 ± 0.48	2.05 ± 0.35			
Obsessive-Compulsive Symptoms	Teaching Intervention Group	2.4 ± 0.39	2.19 ± 0.23	0.882 (0.031)	11.428** (0.172)	2.713 (0.09)
	Comparison Group	2.43 ± 0.34	2.17 ± 0.27			
	Control Group	2.41 ± 0.4	2.26 ± 0.28			
Emotional Instability	Teaching Intervention Group	2.7 ± 0.58	2.38 ± 0.29	2.484 (0.083)	23.069*** (0.295)	7.342** (0.211)
	Comparison Group	2.64 ± 0.37	2.26 ± 0.35			
	Control Group	2.68 ± 0.4	2.45 ± 0.36			
Interpersonal Sensitivity	Teaching Intervention Group	2.2 ± 0.53	2 ± 0.39	0.834 (0.029)	7.842** (0.125)	2.277 (0.76)
	Comparison Group	2.1 ± 0.42	1.9 ± 0.32			
	Control Group	2.16 ± 0.47	2.02 ± 0.39			
Maladjustment	Teaching Intervention Group	1.88 ± 0.57	1.73 ± 0.53	0.419 (0.66)	4.982* (0.83)	0.23*** (8.213)
	Comparison Group	1.82 ± 0.51	1.61 ± 0.33			
	Control Group	1.81 ± 0.7	1.76 ± 0.5			
Psychological Imbalance	Teaching Intervention Group	1.53 ± 0.58	1.54 ± 0.54	0.257 (0.009)	0 (0)	0.19 (0.003)
	Comparison Group	1.46 ± 0.31	1.47 ± 0.25			
	Control Group	1.43 ± 0.5	1.47 ± 0.43			
Academic Stress	Teaching Intervention Group	3.26 ± 0.37	2.67 ± 0.3	3.043 (1)	33.112*** (0.376)	21.323*** (0.437)
	Comparison Group	3.18 ± 0.54	2.76 ± 0.47			
	Control Group	3.22 ± 0.55	2.94 ± 0.54			
Depression	Teaching Intervention Group	2.32 ± 0.3	1.89 ± 0.26	1.101 (0.34)	42.353*** (0.435)	13.094*** (0.323)
	Comparison Group	2.28 ± 0.44	2.03 ± 0.38			
	Control Group	2.25 ± 0.32	2.25 ± 0.33			

*p < 0.05, **p < 0.01, ***p < 0.001.

### 3.3 Correlation analysis between mental health and physical fitness indicators

**Figure 4:**
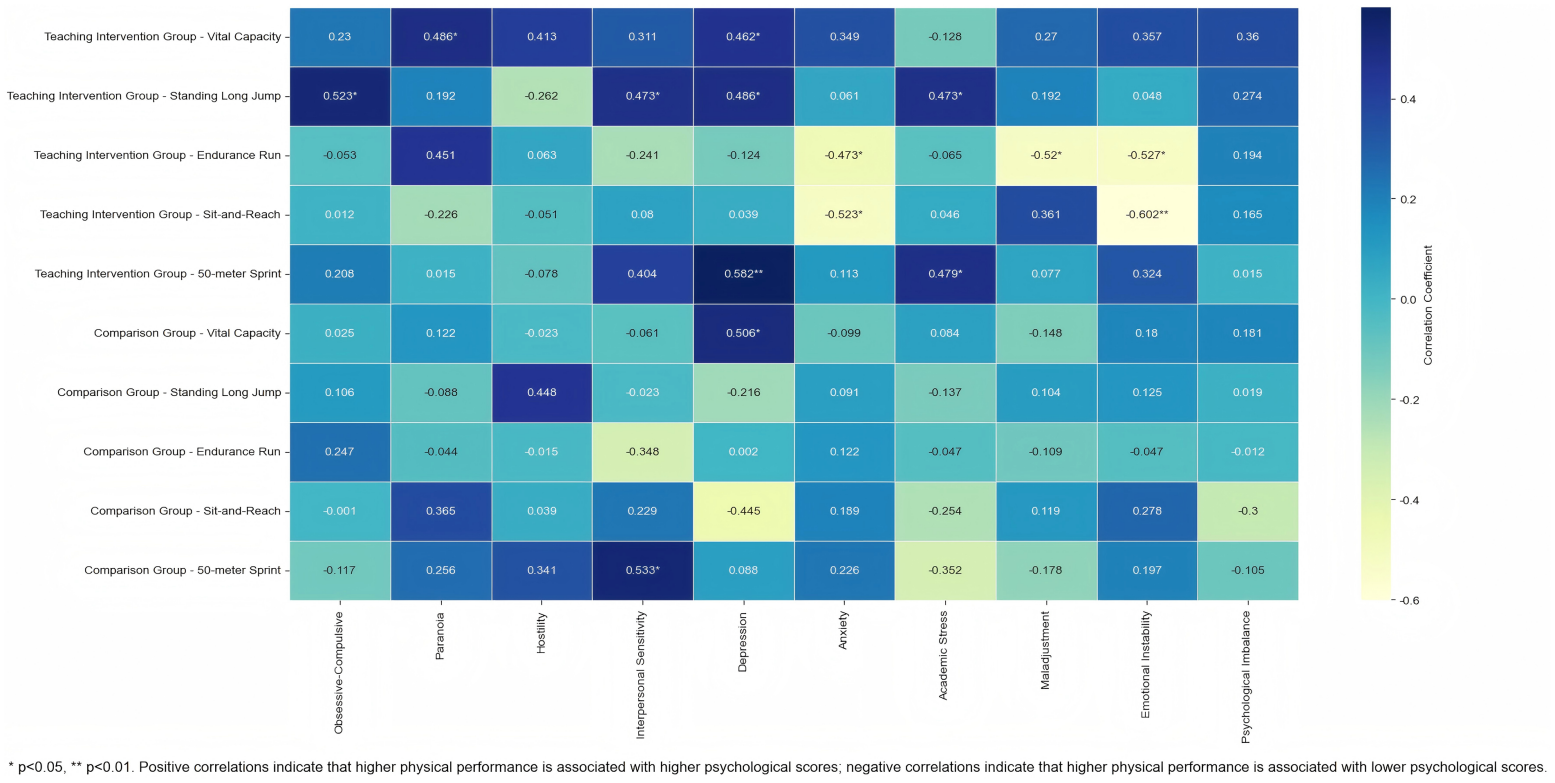
Heatmap of the correlation between mental health and physical fitness indicators.

To further explore the potential associations between physical fitness indicators and dimensions of psychological health, correlation analyses were conducted separately for the Teaching Intervention Group and the Comparison Group. Detailed results are presented in Table 3 and Figure 4.

**Table 3:** Correlation analysis results of various dimensions of mental health and physical fitness indicators.

Group	Indicator	Obsessive-Compulsive	Paranoia	Hostility	Interpersonal Sensitivity	Depression	Anxiety	Academic Stress	Maladjustment	Emotional Instability	Psychological Imbalance
Teaching Intervention Group	Vital Capacity	0.23	0.486*	0.413	0.311	0.462*	0.349	−0.128	0.27	0.357	0.36
Teaching Intervention Group	Standing Long Jump	0.523*	0.192	−0.262	0.473*	0.486*	0.061	0.473*	0.192	0.048	0.274
Teaching Intervention Group	Endurance Run	−0.053	0.451	0.063	−0.241	−0.124	−0.473*	−0.065	−0.520*	−0.527*	0.194
Teaching Intervention Group	Sit-and-Reach	0.012	−0.226	−0.051	0.08	0.039	−0.523*	0.046	0.361	−0.602**	0.165
Teaching Intervention Group	50-meter Sprint	0.208	0.015	−0.078	0.404	0.582**	0.113	0.479*	0.077	0.324	0.015
Comparison Group	Vital Capacity	0.025	0.122	−0.023	−0.061	0.506*	−0.099	0.084	−0.148	0.18	0.181
Comparison Group	Standing Long Jump	0.106	−0.088	0.448	−0.023	−0.216	0.091	−0.137	0.104	0.125	0.019
Comparison Group	Endurance Run	0.247	−0.044	−0.015	−0.348	0.002	0.122	−0.047	−0.109	−0.047	−0.012
Comparison Group	Sit-and-Reach	−0.001	0.365	0.039	0.229	−0.445	0.189	−0.254	0.119	0.278	−0.3
Comparison Group	50-meter Sprint	−0.117	0.256	0.341	0.533*	0.088	0.226	−0.352	−0.178	0.197	−0.105

*p < 0.05, **p < 0.01; Unmarked items indicate no significant correlation. Positive correlation means higher physical fitness indicators correspond to higher mental health dimensions; negative correlation indicates that higher physical fitness indicators are associated with lower mental health dimensions.

In the Teaching Intervention Group, several significant correlations were found between physical fitness indicators and psychological health dimensions. Vital Capacity was significantly and positively correlated with Paranoia (*r* = 0.486, *p* < 0.05) and Depression (*r* = 0.462, p < 0.05), suggesting that individuals with better lung function tended to report higher levels of paranoia and depressive symptoms. Standing Long Jump performance showed significant positive correlations with Obsessive-Compulsive Symptoms (*r* = 0.523, *p* < 0.05), Interpersonal Sensitivity (*r* = 0.473, *p* < 0.05), Depression (*r* = 0.486, p < 0.05), and Academic Stress (*r* = 0.473, p < 0.05).

In contrast, the Endurance Run was negatively correlated with Anxiety (*r* = −0.473, *p* < 0.05), Maladjustment (*r* = −0.520, *p* < 0.05), and Emotional Instability (*r* = −0.527, *p* < 0.05), indicating that higher endurance was associated with lower levels of psychological distress. Similarly, performance on the Sit-and-Reach Test (flexibility) showed significant negative correlations with Anxiety (*r* = −0.523, *p* < 0.05) and Emotional Instability (*r* = −0.602, *p* < 0.01), suggesting a potential link between flexibility and emotional regulation. Notably, 50-meter Sprint time was positively correlated with Depression (*r* = 0.582, *p* < 0.01) and Academic Stress (*r* = 0.479, *p* < 0.05), indicating that poorer sprint performance (i.e., longer sprint time) was associated with greater psychological burden.

In contrast, only two significant correlations were observed in the Comparison Group: Vital Capacity was positively correlated with Depression (*r* = 0.506, *p* < 0.05), and 50-meter Sprint time was positively correlated with Interpersonal Sensitivity (*r* = 0.533, *p* < 0.05). No other significant associations between physical and psychological health indicators were found in this group.

## 4 Discussion and analysis

### 4.1 The impact of aerobic exercise combined with blood flow restriction on physical fitness indicators in high school students

This study investigated the comprehensive effects of aerobic exercise combined with BFR on physical fitness indicators in high school students. The results showed that both the Teaching Intervention Group and the Comparison Group effectively improved adolescents’ overall physical fitness; however, differences emerged in the extent of improvement and underlying physiological mechanisms. Overall, both training approaches positively impacted Vital Capacity, Endurance Run, short-distance speed, lower limb explosive strength, and flexibility. Vital Capacity significantly improved in both the Teaching Intervention Group (aerobic exercise combined with BFR) and the Comparison Group (aerobic exercise only), with comparable magnitudes of improvement. This suggests that both approaches are equally effective in enhancing the endurance and coordination of the respiratory muscles. These findings align with recent research by Sardeshmukh and Gunjal (2023) and Wu et al. (2020), which demonstrated that systematic aerobic training significantly improves respiratory muscle function and pulmonary ventilation efficiency. In addition, Yang et al. (2022) pointed out that BFRT can induce a localized hypoxic environment in the distal limbs, mimicking the physiological effects of high-intensity exercise. This may enhance oxygen transport and diffusion capacity in the lungs, improve respiratory muscle function, and thereby enhance overall aerobic capacity.

Regarding endurance performance, the Teaching Intervention Group showed more pronounced improvements than the Control Group. Prior studies have suggested that BFRT can significantly increase the accumulation of local metabolites such as lactate, triggering metabolic stress responses that facilitate muscular adaptation (Bower, 2021). In this study, the high local metabolic stress induced by BFRT may have contributed to improved lactate tolerance and metabolic adaptability. Although its short-term effects were not markedly superior to aerobic exercise alone, BFRT’s potential for enhancing endurance performance over a longer time frame warrants further attention. Aerobic exercise alone is already effective in improving muscular endurance and cardiopulmonary function within a relatively short duration (Hughes et al., 2018), suggesting that BFRT may require a longer adaptation period to fully exhibit its advantages in endurance enhancement.

In terms of short-distance speed and lower limb explosive strength (Standing Long Jump), both the Teaching Intervention Group and the Comparison Group showed significant improvements, indicating that both training modalities can effectively enhance rapid power output and neuromuscular coordination. However, the Teaching Intervention Group showed a greater improvement in the Standing Long Jump than the Control Group, suggesting a more pronounced advantage in developing explosive power. This effect may be attributed to BFRT’s characteristic localized hypoxia and elevated metabolic stress, which are conducive to recruiting fast-twitch muscle fibers, enhancing lactate tolerance, and improving neuromuscular efficiency (Wang et al., 2023b). By contrast, while aerobic exercise also significantly improves performance, its limited direct stimulation of fast-twitch fibers may render it less effective than BFRT in enhancing explosive strength (Gervasi et al., 2018).

In terms of flexibility (Sit-and-Reach Test), both the Teaching Intervention Group and the Comparison Group demonstrated significant improvements, with the Teaching Intervention Group showing a greater pre–post change. This suggests that BFRT may be more effective in improving flexibility. The underlying mechanism may involve BFRT-induced metabolic stress, which leads to localized tissue edema and metabolite accumulation, thereby increasing local temperature and blood flow, enhancing the extensibility of muscles and fascia (Li et al., 2022). Although aerobic exercise also contributes to flexibility improvement, the lack of large joint range of motion and sustained high-intensity localized stimulation may limit its overall effect (Fett et al., 2009).

In summary, the study confirms that aerobic exercise combined with BFR offers more pronounced benefits in short-distance speed, lower limb explosive strength, and flexibility, while yielding comparable short-term effects to aerobic exercise alone in improving Vital Capacity. Future studies should consider extending the training duration and optimizing training load and recovery strategies to further investigate the long-term effects and safety of BFRT. Importantly, the large effect sizes observed across multiple fitness outcomes indicate not only statistical significance but also practically meaningful benefits, underscoring the translational value of incorporating BFRT into school-based physical education and adolescent health promotion programs.

### 4.2 Discussion on the impact of aerobic exercise combined with blood flow restriction on mental health indicators

This study found that aerobic exercise combined with BFR demonstrated significant advantages in improving psychological health among high school students, particularly in key dimensions such as Anxiety, Depression, and Academic Stress. Although between-group differences for Depression and Academic Stress were not statistically significant, both outcomes exhibited large effect sizes, a pattern often observed in behavioral intervention studies where high within-group variability can mask statistically significant differences despite substantial changes in magnitude (Lakens, 2013). The Teaching Intervention Group showed a large effect size improvement in the Anxiety factor (*d* = 1.51), significantly greater than the Comparison Group (*p* < 0.05, *d* = 1.00), and exceeding the moderate-to-large effect size (*d* = 0.88) reported in the meta-analysis by Huang et al. (2024) on aerobic exercise interventions for anxiety among university students. Improvement in Depression was especially prominent (*d* = 1.72), also surpassing the average effect size (*d* = 0.82) summarized by Bailey et al. (2018) in studies on physical activity interventions for adolescents and young adults. Moreover, the Teaching Intervention Group showed a significant improvement in Academic Stress (*d* = 1.43), a particularly relevant issue for Chinese high school students, suggesting that BFRT may enhance stress coping abilities by inducing moderate physiological stress (Silva et al., 2021). Improvements in Emotional Instability (*d* = 0.70) and Maladjustment (*d* = 0.83) were also observed, further supporting the potential of BFRT in adolescent psychological function restoration. These changes are practically meaningful, as reductions in anxiety, depression, and stress can directly support students’ academic performance, resilience, and long-term well-being.

These prominent effects may be associated with specific neurobiological mechanisms induced by BFRT. Previous studies have demonstrated that BFRT can significantly stimulate the secretion of growth hormone and insulin-like growth factor-1 (Takano et al., 2005), both of which are not only involved in physical recovery but also play important roles in emotional regulation and cognitive function (Karachaliou et al., 2021). Chen et al. (2022) further noted that BFRT induces an acute increase in cortisol levels followed by a rapid decline during recovery. This hormonal fluctuation may enhance adaptation to acute exercise stress via activation of the hypothalamic–pituitary–adrenal (HPA) axis, while helping to maintain a stable testosterone-to-cortisol ratio, thus improving tolerance to long-term training stress. Neuroimaging studies provide additional mechanistic support: Ganesan et al. (2015) found that BFRT can alter oxygenation in the prefrontal cortex, a brain region closely associated with executive function and emotional regulation. Törpel et al. (2018) further confirmed that metabolic stress induced by BFRT significantly increases levels of brain-derived neurotrophic factor (BDNF), a key biomarker of neuroplasticity that is closely related to depressive symptom relief (Zhou et al., 2017). This mechanism may underlie the large effect size observed for the Depression factor in this study.

At the level of emotional regulation and social adaptation, the Teaching Intervention Group also showed a medium effect size improvement in the Hostility factor (*d* = 0.69), suggesting the potential of BFRT to enhance psychological resilience. San Román-Mata et al. (2020) emphasized that moderate and controllable physiological stress contributes to the development of resilience. BFRT, through partial occlusion of limb blood flow and localized metabolic stress, offers a safe and manageable dual challenge—both psychological and physiological—for adolescents (Patterson et al., 2019; De Queiros et al., 2024). For high school students in a critical stage of physical and mental development, repeated exposure to controlled physical discomfort may foster greater self-efficacy and improved coping with social stress. It is important to note that no significant improvements were observed in structural psychological factors such as Paranoia, Obsessive-Compulsive Symptoms, Interpersonal Sensitivity, and Psychological Imbalance. This finding is consistent with existing literature suggesting that personality traits, as relatively stable psychological constructs, are generally resistant to short-term exercise interventions (Zhang et al., 2024). These issues are often shaped by long-term influences such as individual personality, family structure, and social interaction patterns. Without mechanisms for cognitive restructuring or social feedback, single-mode physical interventions are limited in their ability to produce fundamental changes in these domains.

In conclusion, aerobic exercise combined with BFR demonstrated relatively superior intervention effects in addressing core psychological issues such as anxiety, depression, and academic stress in adolescents, highlighting its potential value as a complementary approach to traditional exercise-based mental health interventions. Taken together, these findings highlight that the large effect sizes observed are not merely statistically significant but also carry practical implications, reinforcing the potential of BFRT as a school-based strategy to promote adolescent mental health. Future research should explore the feasibility of integrating BFRT with cognitive behavioral therapy, social skills training, and other multidimensional interventions to expand its applicability in comprehensive psychological care models.

### 4.3 Discussion on the correlation between mental health and physical fitness indicators

This study further explored the potential impact of aerobic exercise combined with BFR on the coupling mechanisms between physical fitness and psychological health in high school students. The results indicated that in the Teaching Intervention Group, ten significant correlations were observed between physical and psychological health indicators, compared to only two in the Comparison Group. This suggests that, relative to conventional aerobic exercise, BFRT may more effectively activate the physiological–psychological coupling response. The difference was not only reflected in the number of significant correlations but also in the direction and underlying regulatory mechanisms of those associations.

In the Teaching Intervention Group, certain physical fitness indicators showed positive correlations with negative psychological dimensions—most notably, Vital Capacity and Standing Long Jump were positively correlated with Paranoia, Depression, Obsessive-Compulsive Symptoms, and Academic Stress. These findings deviate from the traditional linear assumption that improved physical fitness leads to better psychological outcomes. Despite overall improvements in both physical and psychological indicators following the intervention, structural correlations at the post-intervention stage revealed that individuals with higher physical performance sometimes scored higher on certain negative psychological dimensions. This may be related to increased physiological load, elevated performance expectations, or heightened self-imposed pressure during exercise, leading to additional psychological stress (Sorkkila et al., 2020).

Notably, in both the Teaching Intervention Group and the Comparison Group, vital capacity was positively correlated with depressive symptoms, and in the intervention group, it was also positively correlated with paranoia. This pattern contrasts with the inverse association between lung function and psychological distress commonly reported in previous studies, representing a counterintuitive outcome. One possible explanation is that individuals with greater lung capacity often engage in higher-intensity training and competitive activities, which can increase exposure to performance pressures, external evaluations, and perfectionistic tendencies. Such psychosocial demands may contribute to heightened emotional strain. For instance, Gerber et al. (2018) found that higher perceived stress in adolescent athletes was associated with greater burnout and depressive symptoms, and discussed how high training loads might contribute to these outcomes. Hill and Curran (2016) reported that perfectionism can function as a double-edged sword in sport, being linked to both high athletic performance and increased vulnerability to psychological distress. These findings suggest that, in specific contexts, superior physical capacity may coexist with greater psychological strain rather than unilaterally promoting mental health. Additionally, the dual burden of pursuing both athletic excellence and academic achievement may create compensatory stress, further exacerbating psychological strain in high-performing students. Given the cross-sectional nature of this study and the possibility of unmeasured confounders, these interpretations should be viewed as tentative, and future longitudinal and experimental studies are warranted to verify them.

From a physiological perspective, BFRT induces hypoxic stress and metabolic byproduct accumulation by restricting venous return, potentially activating the sympathetic nervous system and increasing cortisol levels (Pope et al., 2013),which may not only facilitate physical adaptation but also contribute to psychological responses such as heightened emotional tension or mood disturbances. Given that adolescents’ nervous systems are still in development, this physiological stress may trigger maladaptive psychological responses in some individuals, such as heightened anxiety or mood fluctuations—externalizing and internalizing behavioral issues often seen during adolescence (Poon et al., 2016). Furthermore, some individuals may possess strong physical adaptability but limited psychological regulatory capacity. This type of “edge-value effect” may be amplified statistically, emphasizing the importance of accounting for individual differences in adolescent intervention studies.

At the same time, a series of negative correlations observed in the Teaching Intervention Group aligned closely with existing literature. Indicators such as Endurance Run and Sit-and-Reach Test were negatively correlated with Anxiety, Emotional Instability, and Maladjustment. This suggests that students with better cardiorespiratory endurance and flexibility tended to maintain more stable emotions and stronger adaptive capacities in class. These findings are consistent with those of Tian et al. (2025) and Li et al. (2024), which confirmed that aerobic exercise can alleviate depressive symptoms and enhance emotional regulation through increased levels of brain-derived neurotrophic factor (BDNF) and *β*-endorphins, enhancing neuroplasticity and functional connectivity in related brain regions. The intervention model used in this study combined BFRT for enhancing physical performance with aerobic exercise, which may have played a key role in psychological modulation. Together, these elements contributed to a synergistic improvement in both physical and mental health.

In contrast, the correlation structure in the Comparison Group appeared more fragmented, with only two significant positive associations observed: between Vital Capacity and Depression, and between 50-meter Sprint time and Interpersonal Sensitivity. These isolated findings lacked systematic consistency and directionality. This result aligns with the viewpoint of Alexander and Machado (2024), who suggested that without structured intervention and sufficient training stimulus, exercise may fail to activate stable regulatory pathways between brain and body systems, thereby limiting its psychological benefits.

In summary, this study revealed that aerobic exercise combined with BFR not only improves high school students’ physical and psychological health independently but may also activate a stronger coupling mechanism between the two domains. The observed multidimensional relationship between physical improvement and psychological adaptation offers a novel intervention framework for school-based physical education. Although some nonlinear correlations emerged—highlighting the need to consider differential psychological responses to higher training intensities—the overall trend supports the notion that a well-integrated combination of BFR and aerobic exercise can facilitate coordinated physical and psychological development. Future instructional design may benefit from dynamically adjusting training strategies based on individual characteristics to further maximize the mental health benefits of physical fitness improvements.

## 5 Conclusion

This study demonstrated that aerobic exercise combined with BFR significantly improves both physical fitness and psychological health in high school students. Compared to aerobic exercise alone, this integrated training approach yielded superior effects in enhancing endurance, speed, strength, and flexibility, while also proving more effective in alleviating core psychological issues such as anxiety, depression, and academic stress. Moreover, the results suggest that this intervention promotes a synergistic coupling between physiological and psychological functions, supporting the coordinated development of adolescent physical and mental well-being. As a safe and feasible instructional strategy, BFRT holds strong potential for implementation in school-based physical education, providing theoretical and practical support for the development of systematic and evidence-based youth health promotion programs.

### 5.1 Limitations

This study has several limitations. First, the sample size was relatively small, and all participants were recruited from a single school, which may limit the generalizability of the findings. Second, the 12-week intervention period did not include long-term follow-up, making it difficult to assess the sustainability of the intervention effects. Third, the study did not employ a double-blind design, and psychological health assessments were primarily based on self-reported measures, which may introduce subjective bias. However, standardized administration procedures and assurances of confidentiality were implemented to reduce this risk. Future research should consider expanding the sample size, extending the follow-up duration, and incorporating objective physiological indicators to better elucidate the underlying mechanisms and verify the long-term efficacy of the intervention.

## Data Availability

The raw data supporting the conclusions of this article will be made available by the authors, without undue reservation.
